# Effects of Platelet-Rich Plasma on the Oxymetholone-Induced Testicular Toxicity

**DOI:** 10.3390/diseases11020084

**Published:** 2023-06-09

**Authors:** Amal I. Saba, Reda H. Elbakary, Omayma K. Afifi, Heba E. M. Sharaf Eldin

**Affiliations:** Histology and Cell Biology Department, Faculty of Medicine, Tanta University, Tanta 31527, Egypt

**Keywords:** oxymetholone, platelet-rich plasma, albino rat, testis, therapeutic effects, histology investigation

## Abstract

Oxymetholone is one of the anabolic steroids that has widely been used among teenagers and athletes to increase their muscle bulk. It has undesirable effects on male health and fertility. In this study, the therapeutic effects of platelet-rich plasma (PRP) on oxymetholone-induced testicular toxicity were investigated in adult albino rats. During the experiments, 49 adult male albino rats were divided into 4 main groups: Group 0 (donor group) included 10 rats for the donation of PRP, Group I (control group) included 15 rats, Group II included 8 rats that received 10 mg/kg of oxymetholone orally, once daily, for 30 days, and Group III included 16 rats and was subdivided into 2 subgroups (IIIa and IIIb) that received oxymetholone the same as group II and then received PRP once and twice, respectively. Testicular tissues of all examined rats were obtained for processing and histological examination and sperm smears were stained and examined for sperm morphology. Oxymetholone-treated rats revealed wide spaces in between the tubules, vacuolated cytoplasm, and dark pyknotic nuclei of most cells, as well as deposition of homogenous acidophilic material between the tubules. Electron microscopic examination showed vacuolated cytoplasm of most cells, swollen mitochondria, and perinuclear dilatation. Concerning subgroup IIIa (PRP once), there was a partial improvement in the form of decreased vacuolations and regeneration of spermatogenic cells, as well as a reasonable improvement in sperm morphology. Regarding subgroup IIIb (PRP twice), histological sections revealed restoration of the normal testicular structure to a great extent, regeneration of the spermatogenic cells, and most sperms had normal morphology. Thus, it is recommended to use PRP to minimize structural changes in the testis of adult albino rats caused by oxymetholone.

## 1. Introduction

Over recent decades, the effects of environmental pollutants, endocrine disruptors, and pharmaceutical products on different human organisms and systems (including the reproductive system) have received more and more attention. These pollutants and chemicals may cause reproductive toxicity, which has serious effects on fertility and sexual function of human beings [1]. 

Oxymetholone is one of the anabolic steroids that has been increasingly used among athletes and teenagers to increase their muscle bulk. It is also used in the treatment of certain types of anemia and osteoporosis and stimulates muscle growth in malnourished patients [2]. Furthermore, oxymetholone activates androgen receptor-mediated signaling, which stimulates the production of erythropoietin and the synthesis of protein. Despite the aforementioned advantages of oxymetholone, it has many adverse effects, such as jaundice, elevated hepatic enzymes, hyperlipidemia, sodium retention, hyperchloremia, edema, hypercalcemia, hyperphosphatemia, and excitability. Additionally, it has serious side effects on the testis, such as testicular atrophy, inhibition of testicular function, oligospermia, epididymitis, and chronic priapism [3].

Some studies demonstrated that oxymetholone was associated with oxidative stress and cellular damage in many organs, particularly the kidney and the liver [4], as well as inducing disturbances in the testicular enzymes and hormones [5]. 

Platelet-rich plasma (PRP) is a blood-derived product that contains high platelet concentrations which are more than five times that in the whole blood [6]. PRP contains a unique composition of cytokines and growth factors, including vascular endothelial growth factor (VEGF), fibroblast growth factor (FGF), platelet-derived growth factor, interleukin-1B, interleukin-10, insulin-like growth factor-1, and tumor necrosis factor-B [7]. PRP has many beneficial effects, mediated by these growth factors, such as accelerated angiogenesis, anabolism, and anti-inflammatory and regenerative effects. Its applications in treating muscle and skeletal injuries and enhancing hair re-growth have dramatically increased nowadays [8]. 

Moreover, PRP has been used in treating diseases of other organs, such as the ovary [9,10,11], prostate [12,13,14], postpartum perineal repair failure [15], musculoskeletal system [16], and lumbar disc herniation [17]. Additionally, previous studies have proven the beneficial effects of PRP on testes in the ischemia-reperfusion model [18] and the testicular torsion model [19]. 

Keeping in mind the deleterious effects of oxymetholone on the reproductive health of human beings, this work aimed at detecting the effect of oxymetholone on the testis of adult albino rats and innovatively evaluating the possible therapeutic role of PRP.

## 2. Materials and Methods

### 2.1. Chemicals

Oxymetholone was obtained from El-Gomhoria Company for Trading Medical Appliances and Pharmaceutical Chemicals, Tanta, Egypt, in the form of tablets. Its trade name is Anadrol 50.

### 2.2. Animals

**Figure 1:**
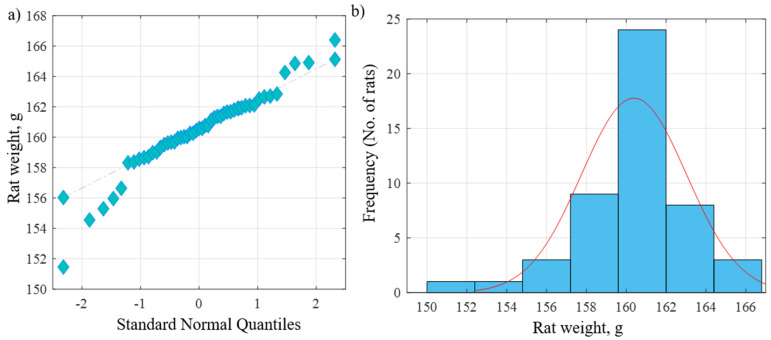
(**a**) QQ plot of the sample data versus normal standards. (**b**) Histogram of the rats’ weights at the beginning of the experiment.

**Figure 2:**
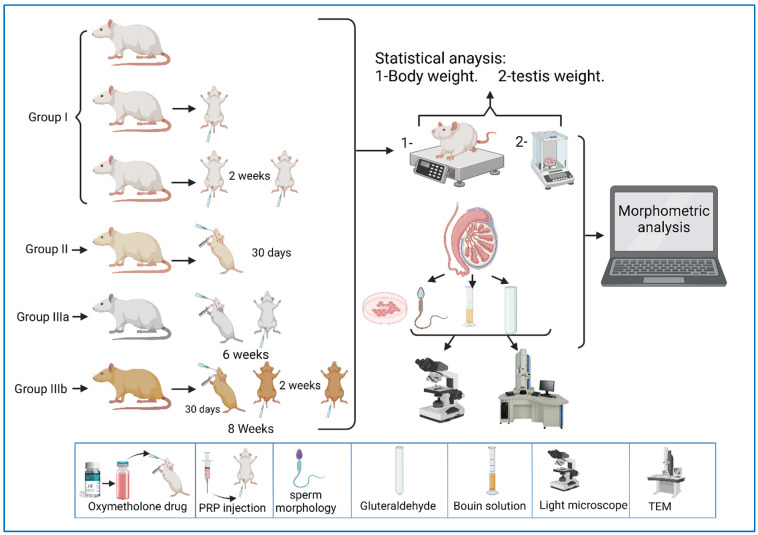
A representative diagram of the experimental design.

The current study was carried out on 49 adult male albino rats. All adult rats were handled by the use/care guide of laboratory animals [20]. The study was executed after receiving the approval of the ethics committee at the Faculty of Medicine, Tanta University, Tanta, Egypt (approval number: 33728/03/20). The weight of the experimental rats varied between 151.46 and 166.39 g, with an average weight of 160.38 g and a standard deviation of 2.64 g. Figure 1a shows the QQ plot of the sample data versus normal standards. Figure 1b shows the histogram of the rats’ weight at the beginning of the experimentation. It was observed that most of the rats had initial weights of around 160 g, whereby 24 rats had an initial weight in the range of 160–162 g. There were a few rats (less than five rats) with an outlier weight (less than 154 g or more than 164 g). Rats were housed in properly ventilated and clean cages under the same environmental conditions and fed on a similar laboratory diet. All rats were acclimatized for a week. They were classified as follows (Table 1 and Figure 2):

Group 0 (donor group): consisted of 10 rats to prepare for the donation of blood (PRP).

Group I (control group): included 15 rats that were equally subdivided into: subgroup Ia, that were left untreated up to the end of the experiment, subgroup Ib, which were treated with 0.1 mL of PRP once, locally in the testis [21], and sacrificed after two weeks, and subgroup Ic, which were treated with PRP (two injections, with a two-week interval between each injection) and sacrificed after two weeks of PRP injections. 

Group II (oxymetholone group): consisted of 8 rats that received oxymetholone (10 mg/kg) once daily, orally, for 30 days, and were then sacrificed [22]. 

Group III (PRP-treated group): consisted of 16 rats that were subdivided into two equal subgroups:

Subgroup IIIa (oxymetholone + a single dose of PRP): received oxymetholone by the same dose, route, and time of administration as group II, and were then treated once with a 0.1 mL intratesticular dose of PRP the day after the last oxymetholone dose, and were sacrificed after two weeks of PRP injection. 

Subgroup IIIb (oxymetholone + two doses of PRP): received oxymetholone by the same dose, route, and time of administration as group II, then PRP was injected the day after the last oxymetholone dose, two times, in two-week intervals (0.1 mL per injection), and the rats were sacrificed two weeks after the last PRP injection.

### 2.3. Preparation of PRP

PRP was prepared according to the following procedures [18,23]. 

Blood was collected from donor rats (group 0) by cardiac puncture after being anesthetized by ether inhalation. PRP was prepared by collecting 3 mL of blood from each rat using a sterile syringe containing 0.3 mL of sodium citrate and transferring it to a sterile falcon tube. The citrated blood was centrifuged at 3000 rpm for 7 min and the supernatant, which contains the buffy coat with platelets and leucocytes, was aspirated by a micropipette, and then it was placed in another sterile falcon tube for a second centrifugation at 4000 rpm for 5 min. Platelet pellets appeared in the bottom of the tube, which represents PRP. The platelet pellets were resuspended in 1 mL of the supernatant, representing the platelet-poor plasma (PPP). Finally, 0.1 mL of PRP was immediately injected into each rat’s testis by an insulin syringe. 

At the end of the experiment, the rats were weighed, anesthetized, and sacrificed. Both testes from each animal were dissected, and the weight of each was estimated; then, the right testis was processed for the electron microscopic study and the left was processed for histological and histochemical preparation. A sample was obtained from the epididymis of each animal and smears were stained by eosin for histological examination of sperm morphology [24].

### 2.4. Histological/Histochemical Study

#### 2.4.1. Specimens’ Preparation for Light Microscopic Examination [25]

Fixation of testicular specimens was performed in Bouin’s solution for 24 h. Sections of 5 µm in thickness were cut by the microtome for the histological study [26]. All sections were stained using the following methodology.

Hematoxylin and eosin (H-E) stains [25] were used to demonstrate the general histological features, while the periodic acid–Schiff (PAS) technique allowed to show mucopolysaccharides in the testis.

#### 2.4.2. Semen Sample Preparation for Light Microscope

Drops of the stained sperm (1–2 drops) were located approximately 1 cm from the edge of a clean microscopic slide. The second slide was gently attached to the slide’s long edge to touch the width of the first slide and pulled across to make a sperm smear. Two to five slides were developed based on the aforementioned procedures from each sperm suspension to confirm any preparation artifacts [24]. 

Then, the slides stained with eosin and the sperm morphology were examined and photographed using an Olympus light microscope (Tokyo, Japan) coupled to an Olympus digital camera (DXC1850P, Tokyo, Japan) at the Histology and Cell Biology Department, Faculty of Medicine, Tanta University.

#### 2.4.3. Testicular Specimens’ Preparation for Electron Microscope Examination

Specimens were divided into small pieces of 1 mm^3^ in size by a sharp razor blade. They were subjected to the steps for the preparation of semi-thin and ultrathin sections [27]. The specimens were examined using a JEOL-JEM-100 SX electron microscope (Tokyo, Japan) at The Electron Microscopic Unit, Faculty of Medicine, Tanta University.

#### 2.4.4. Morphometric Investigation and Statistical Analysis

Body weight and testicular weight were statistically analyzed. The H&E images of the different experimental groups were examined and processed using Image J software to measure the height of germinal epithelium of all groups in µm, at a magnification of 400, in 10 non-overlapping fields from 10 slides of each rat. The collected data were analyzed using analysis of variance (ANOVA) considering the one-way technique, followed by Tukey’s technique to find the differences between different groups and the control groups [28]. 

#### 2.4.5. Preparation Methodology for Sperm Morphology

The area of the epididymis near the vas deferens was stabbed, and gentle pressure was applied to expose the epididymal contents; then, sections of the epididymis were removed and placed in normal saline, allowing the sperm to swim out. The epididymis was cut into small pieces in 4 mL of saline. The epididymal content was placed in a sterile tube. Centrifugation was performed at 500 rpm for approximately 3 min. Fat rose to the top, debris sank, and a suspension of sperm was left in the middle [24].

The samples used in the smear preparation were stained with eosin to help with visualization of the sperms. Here, 1 mL of sperm suspension was placed into a test tube; then, two drops of 1% eosin Y were put into the test tube and then mixed by gentle agitation. Sperms were incubated at room temperature for about 45–60 min to allow for staining and then resuspended with a Pasteur pipette. 

Slides were washed in water, followed by alcohol, and dried well before use. Samples were mixed and the smear was prepared to avoid potential damage to the sperm, and then air-dried smears were used [29]. 

The method used was a modification of the blood smear preparation. One to two drops of the stained sperm suspension were placed approximately 1 cm from the edge of a precleaned microscopic slide placed on a flat surface. A second slide was held in the right hand, with the slide’s long edge gently touching across the width of the sperm slide, and it was then pulled across to make a sperm smear. Slides were prepared from each sperm suspension. The slides were examined to detect any abnormalities in the morphology of the sperm and photographed using an Olympus light microscope (Tokyo, Japan) coupled to an Olympus digital camera (DXC1850P, Tokyo, Japan), at the Histology and Cell Biology Department, Faculty of Medicine, Tanta University.

## 3. Results 

No mortality was recorded throughout the experimental period. 

### 3.1. Light Microscopic Results

#### 3.1.1. Hematoxylin and Eosin

**Figure 3:**
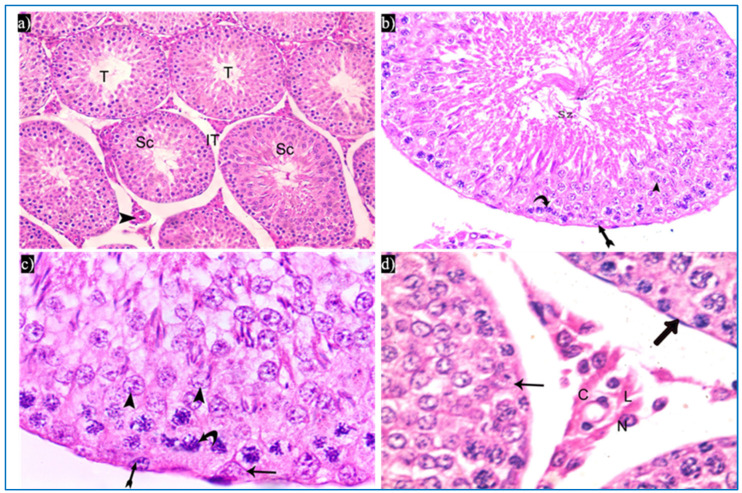
Photomicrographs of the testis of the control group, showing: (**a**) Rounded to oval seminiferous tubules (T) lined by spermatogenic cells (Sc) and separated by the interstitial tissue (IT) with blood vessels in between (arrowhead). H&E, ×200. (**b**) The seminiferous tubule, lined by spermatogenic cells, including spermatogonia (bifid arrow), primary spermatocytes (curved arrow), and spermatids (arrowhead), spermatozoa (Sz). H&E ×400. (**c**) Spermatogonia (bifid arrow), primary spermatocytes (curved arrow), spermatids (arrowheads), and Sertoli cells (arrow). H&E ×1000. (**d**) Leydig cells (L) with rounded nuclei, prominent nucleoli (N), and slightly vacuolated acidophilic cytoplasm (C), and myoid cells (thick arrow) and Sertoli cells, with a pyramidal nucleus and prominent nucleolus (arrow). H&E ×1000.

Sections obtained from the control group revealed the normal histological structure of the testis. Each testis was formed of closely packed seminiferous tubules with regular outlines, and interstitial tissue containing Leydig cells as well as blood vessels in between. Each tubule was lined by germinal epithelium, which is a complex stratified epithelium showing different stages of spermatogenesis (Figure 3a). The spermatogenic cells were arranged from the basal compartment towards the lumen of the tubules among Sertoli cells, and were, namely, spermatogonia, spermatids, spermatozoa, and primary spermatocytes. Spermatids were present in two to four rows near the lumen of the tubules. Early spermatids were rounded, with spherical central pale nuclei having granular chromatin and found near the base of the tubules. Variable numbers of mature sperms were present in the lumen of the tubules (Figure 3b,c). The interstitial tissue contained Leydig cells with rounded nuclei and prominent nucleoli (Figure 3d). 

**Figure 4:**
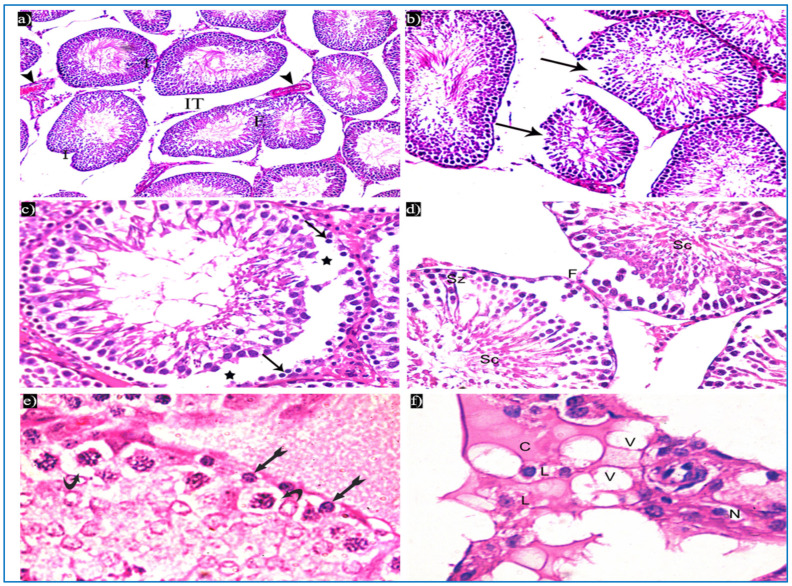
Photomicrographs of a rat testis of group II, showing: (**a**) Irregular outer boundaries (I), fused tubules (F), widely separated seminiferous tubules (IT), and dilated congested blood vessels (arrowheads). H&E, ×200. (**b**) Loss of outer boundaries of the tubules (arrows). H&E, ×200. (**c**) The separation between the basal layer and other spermatogenic cells (*), basal germ cells with very dark nuclei (arrows). H&E, ×400. (**d**) Deeply inserted spermatozoa in between spermatogenic cells (Sz), desquamated spermatogenic cells in the lumen (Sc), fused outer boundaries of two tubules (F). H&E, ×400. (**e**) Vacuolated spermatogonia (bifid arrows) and vacuolated primary spermatocytes (curved arrows). H&E, ×1000. (**f**) Vacuolated interstitial tissue (V) containing Leydig cells (L) with perinuclear vacuolation (N) and highly acidophilic cytoplasm (C). H&E, ×1000.

In the oxymetholone-treated group, disturbances in the normal histological structure of the testis were seen. Some of the seminiferous tubules showed abnormal shapes with indentation and irregularity of their outline, with a fusion of some tubules, and dilated congested blood vessels were also seen in between the tubules (Figure 4a). Partial loss of the outer boundaries was seen in some seminiferous tubules (Figure 4b). A separation between the basal layer of the germinal epithelium and the other spermatogenic cells was noticed, in addition to the basal germ cells showing loss of their cytoplasm with very dark nuclei (Figure 4c). Deeply inserted spermatozoa in between the spermatogenic cells, desquamated spermatogenic cells in the lumen, and fused outer boundaries of the two seminiferous tubules were seen (Figure 4d). In addition, vacuolated spermatogonia and primary spermatocytes were also observed (Figure 4e). Regarding the interstitial tissue, it was homogenous acidophilic, containing many vacuoles, and Leydig cells had perinuclear vacuolation and highly acidophilic cytoplasm (Figure 4f).

**Figure 5:**
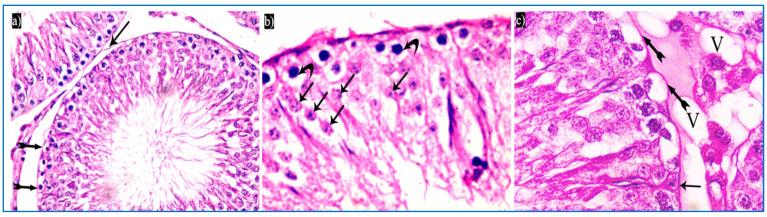
Photomicrographs of a rat testis of subgroup IIIa, showing: (**a**) Spermatogonia with dense nuclei and vacuolated cytoplasm (bifid arrows), and partial detachment of the outer boundary of the tubule (arrow). H&E, ×400. (**b**) Primary spermatocytes with vacuolated cytoplasm (curved arrows), and dark acidophilic cells with fragmented nuclei (arrows). (**c**) Preserved interstitial cells with some vacuoles (V), Sertoli cells with pyramidal elongated nuclei and prominent nucleoli (arrow), and vacuolated spermatogonia (bifid arrows) (b&C, H&E, ×1000).

Subgroup IIIa (received single PRP) showed partial preservation of the normal structure of the seminiferous tubules with the regeneration of the germinal epithelium. A partial detachment of the outer boundary of the tubule was seen, and a few spermatogonia had dark nuclei and cytoplasmic vacuolation (Figure 5a). Vacuolated primary spermatocytes were also observed, and the tubules were filled with dark acidophilic cells with fragmented nuclei (Figure 5b). Preserved Sertoli cells with pyramidal elongated nuclei and prominent nucleoli, and interstitial cells with a few vacuoles, were also seen (Figure 5c).

**Figure 6:**
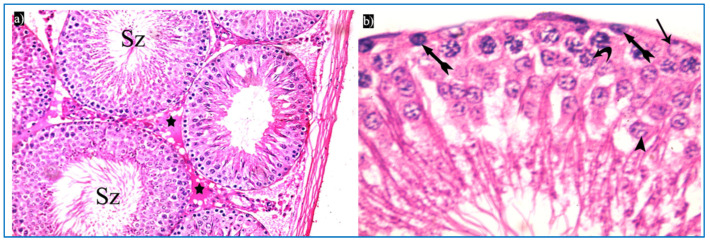
Photomicrographs of a rat testis of subgroup IIIb: (**a**) Spermatozoa in the lumen of the tubules (Sz), and homogenous acidophilic material (*). H&E, ×400. (**b**) Spermatogonia (bifid arrows), primary spermatocyte (curved arrow), early rounded spermatid (arrowhead), Sertoli cells (arrow). H&E, ×1000.

Concerning subgroup IIIb (received two PRP injections), they showed a histological structure of the testes more or less the same as in the control group. They revealed regular seminiferous tubules filled with spermatozoa and normal interstitial tissue in between (Figure 6a). Normal spermatogenic cells and Sertoli cells were also observed (Figure 6b).

#### 3.1.2. Periodic Acid-Schiff’s Reaction (PAS)

**Figure 7:**
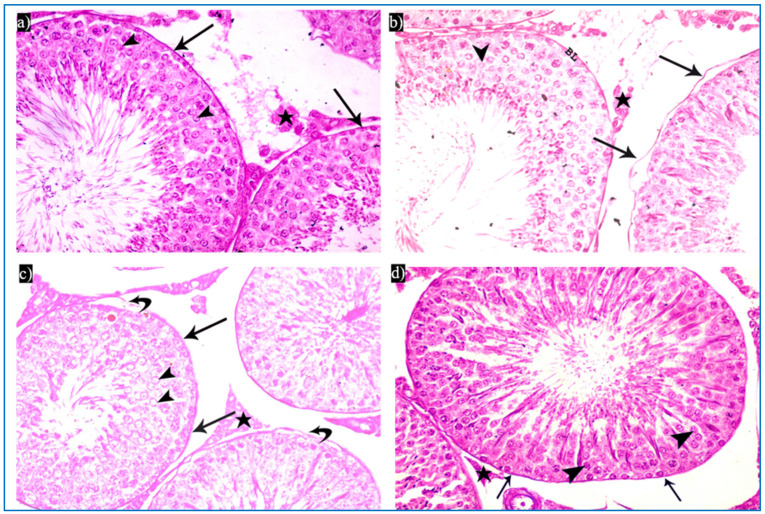
Photomicrographs of rat testis from different sections showing the PAS reaction: (**a**–**d**) basal lamina (arrow), acrosomal cap (arrowhead), and interstitial tissue (*). PAS × 400.

Sections of the control group showed an apparent strong PAS-positive reaction in the basal lamina of the seminiferous tubules, acrosomal caps of spermatids, as well as in the interstitial tissue in between the seminiferous tubules (Figure 7a).

In the oxymetholone group, sections showed an apparent weak PAS-positive reaction in basal lamina, either intact or separated from the germinal epithelium, as shown in Figure 7b. The group that received a single PRP injection revealed an apparent moderate increase in the PAS-positive reaction in the basal lamina of the seminiferous tubules, acrosomal cap of spermatids, and the interstitial tissue in between the tubules (Figure 7c). After two injections of PRP, sections showed an apparent strong PAS-positive reaction in the basal lamina of the seminiferous tubules, acrosomal caps of spermatids, and the interstitial tissue (Figure 7d).

#### 3.1.3. Sperm Morphology Results

**Figure 8:**
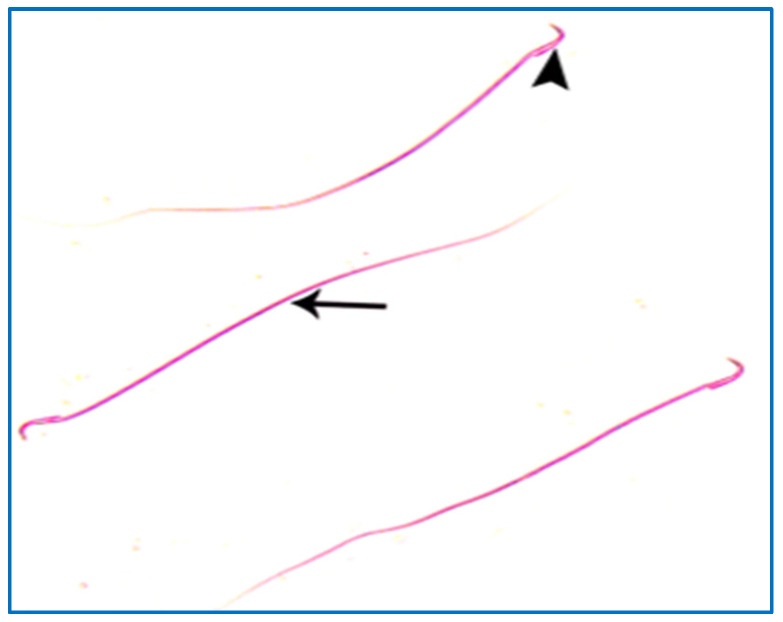
A photomicrograph of rat sperms from group I, showing a head with a characteristic hook (►) and a tail (→) (Eosin, ×1000).

**Figure 9:**
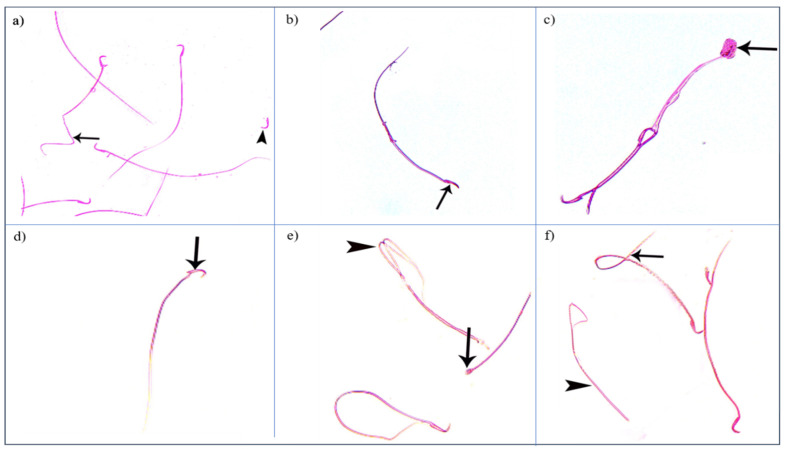
Photomicrographs showing some sperm abnormalities of (group II): (**a**) a head without a tail (►) and an angulated tail (→), (**b**) a hookless head (→), (**c**) a ballooned head (→), (**d**) a head with a hook at the wrong angle (→), (**e**) a pin-shaped head (→) with a double tail (►), (**f**) a tail without a head (►), and a coiled tail (→) (Eosin, ×1000).

Control groups showed normal sperm morphology: each was formed of a characteristic hook-shaped head and a long regular tail, without any coiling or kinking (Figure 8). Concerning the oxymetholone-treated group, some head abnormalities appeared, such as a head without a tail, a hookless head, a ballooned head, a head with a hook at the wrong angle, and a pin-shaped head. The tail abnormalities were represented as a double tail, a tail without a head, and a coiled tail (Figure 9).

**Figure 10:**
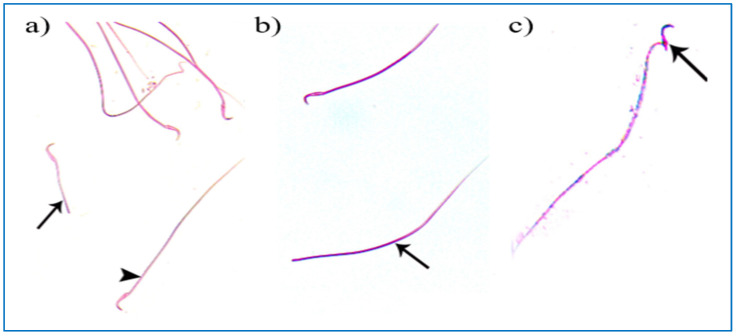
Photomicrographs of rat sperms from subgroup IIIa showing a few sperm abnormalities of the single PRP group: (**a**) an apparently short tail (→) and normal sperm morphology (►), (**b**) a tail without a head (→), and (**c**) a separated head fused with a head of another sperm (→) (Eosin, ×1000).

**Figure 11:**
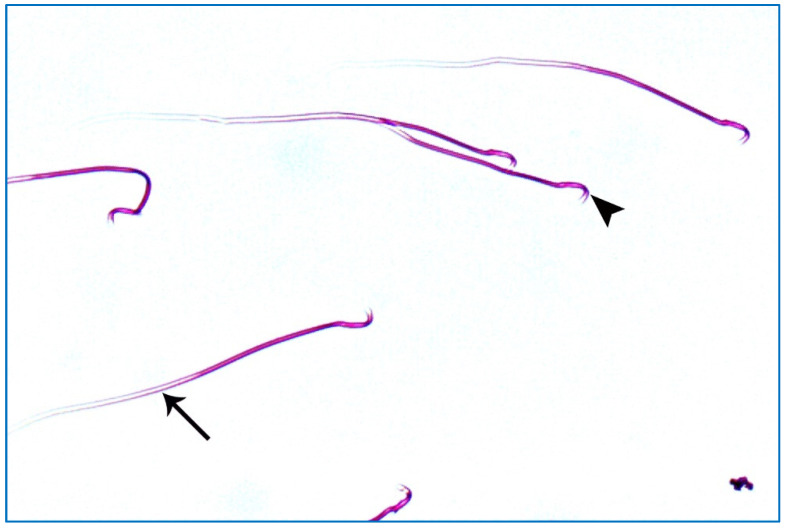
A photomicrograph of rat sperms from subgroup IIIb showing sperms with a nearly normal characteristic head (►) and a long tail (→) (Eosin, ×1000).

Regarding the PRP groups, a few sperm with little abnormalities were observed in subgroup IIIa, in the form of a short tail, a tail without a head, and a separated head fused with a head of another sperm, as shown in Figure 10. On the other hand, in subgroup IIIb, most sperms had normal morphology with characteristic heads and tails, as shown in Figure 11.

#### 3.1.4. Electron Microscopic Results

**Figure 12:**
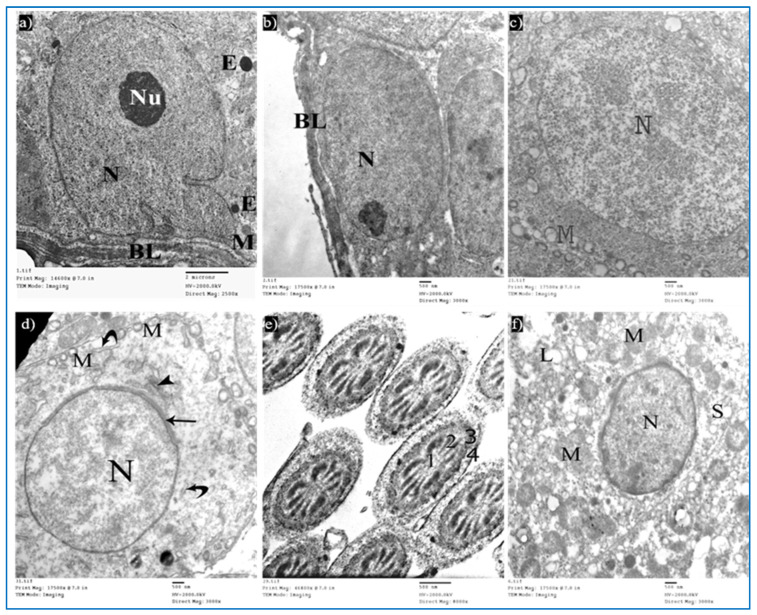
Electron micrographs of a rat of the control group, showing: (**a**) A Sertoli cell resting on basal lamina (BL), nucleus (N) with prominent nucleolus (Nu), mitochondria (M), and electron-dense bodies (E). Mic. Mag. 2500×. (**b**) Type A spermatogonium, with normal nucleus (N), basal lamina (BL). Mic. Mag. 3000×. (**c**) Primary spermatocyte, nucleus (N), characteristic vacuolated mitochondria (M). Mic. Mag. 3000×. (**d**) Spermatid, acrosomal cap (arrow), Golgi apparatus (arrowhead), nucleus (N), mitochondria (M), RER (curved arrows). Mic. Mag. 3000×. (**e**) Middle pieces of spermatozoa tails showing an axoneme (1), nine outer dense fibers (2), circumferentially arranged mitochondria (3), and a flagellar membrane (4). Mic. Mag. 8000×. (**f**) Leydig cell with rounded nucleus (N), SER (S), lipid droplets (L), and normal mitochondria (M). Mic. Mag. 3000×.

Sections of the control group revealed seminiferous tubules containing Sertoli cells and spermatogenic cells. Sertoli cells had large, indented nuclei, with a prominent nucleolus. The cytoplasm contained mitochondria electron-dense bodies (Figure 12a). Type A spermatogonia were resting on the basal lamina and had oval nuclei with granular chromatin (Figure 12b). Primary spermatocytes revealed rounded to oval nuclei with fine chromatin, and their cytoplasm showed multiple rounded to oval mitochondria with a characteristic vacuolated appearance (Figure 12c). Early rounded spermatids were detected by their characteristic acrosomal caps. Their nuclei were rounded with finely granular chromatin and their cytoplasm contained mitochondria arranged regularly at the periphery of the cell (Figure 12d). Middle pieces of the tails of spermatozoa (Figure 12e) and Leydig cells with rounded nuclei were seen. Their cytoplasm contained mitochondria, SER, and lipid droplets (Figure 12f).

**Figure 13:**
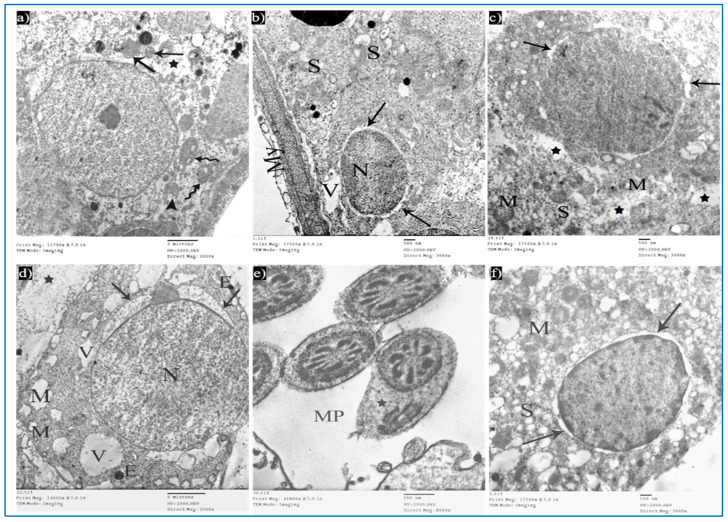
Electron micrographs of a rat of the oxymetholone group, showing: (**a**) A Sertoli cell with an area of cytoplasmic loss (*), normal mitochondria (arrows) or partially vacuolated (arrowhead), and secondary lysosomes (zigzag arrows). Mic. Mag. 2000×. (**b**) Type A spermatogonium, nucleus (N), perinuclear dilatation (arrows), vacuolated cytoplasm (V), myoid cell (My), and dilated SER of neighboring cells (S). Mic. Mag. 3000×. (**c**) Primary spermatocyte dilated perinuclear spacing (arrows), mitochondria (M), cytoplasmic vacuoles (*). Mic. Mag. 3000×. (**d**) Spermatid, acrosomal cap (arrows), nucleus (N), mitochondria (M), vacuoles (V), electron-dense bodies (E), spaces between rounded spermatid and adjacent cells (*). Mic. Mag. 2500×. (**e**) Middle pieces (MP) with excess residual cytoplasm (*). Mic. Mag. 8000×. (**f**) Leydig cells with dilated perinuclear spaces (arrows), destructed mitochondria (M), and dilated SER (S). Mic. Mag. 3000×.

Sections of the oxymetholone group revealed alterations in the structure of Sertoli and spermatogenic cells. Sertoli cells showed a rarified cytoplasm, areas of cytoplasmic loss, degenerated mitochondria, and many secondary lysosomes (Figure 13a). As regards type A spermatogonia, they showed dilated perinuclear cisternae, and the cytoplasm contained multiple vacuoles (Figure 13b). Primary spermatocytes showed cytoplasmic vacuoles, swollen mitochondria, and dilated perinuclear space (Figure 13c). Some early rounded spermatids appeared with dilated acrosomal caps, vacuolated cytoplasm, and swollen mitochondria, and some electron-dense bodies and wide spaces between rounded spermatids and adjacent cells were noticed (Figure 13d). Moreover, middle pieces with excess residual cytoplasm were observed (Figure 13e). Concerning Leydig cells, they showed dilated perinuclear space (Figure 13f). 

**Figure 14:**
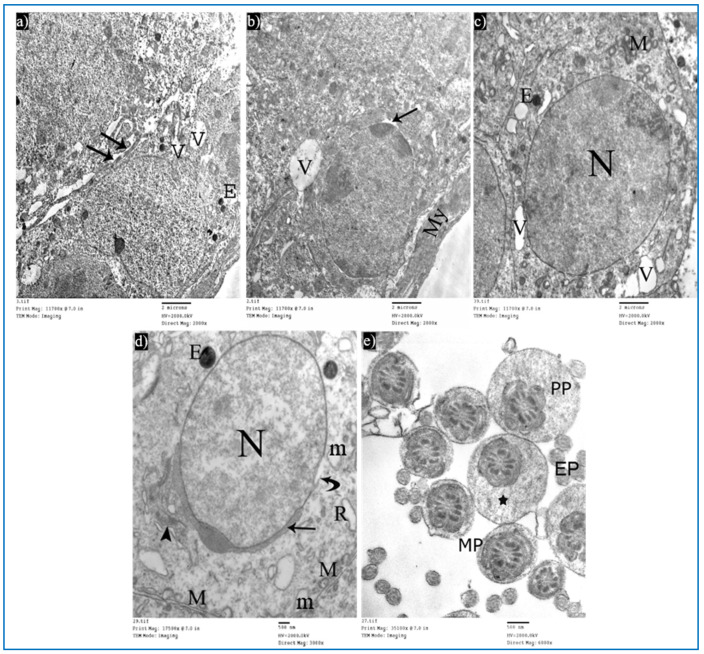
Electron micrographs of a rat of subgroup IIIa, showing: (**a**) A Sertoli cell with electron-dense bodies (E) and vacuoles (V). Mic. Mag. 2000×. (**b**) Type A spermatogonium, myoid cells (My), dilated perinuclear space (arrow), and cytoplasmic vacuole (V). Mic. Mag. 2000×. (**c**) Primary spermatocyte, nucleus (N), mitochondria (M), electron-dense bodies (E), and vacuoles (V). Mic. Mag. 2000×. (**d**) Round spermatid, normal acrosomal cap (arrow), Golgi apparatus (arrowhead), nucleus (N), focal discontinuous nuclear membrane (curved arrow), normal mitochondria (M), swollen mitochondria (m), and dilated RER (R). Mic. Mag. 3000×. (**e**) Middle pieces (MP) and principal pieces (PP) with excess residual cytoplasm (*). Mic. Mag. 6000×.

Sections obtained from testes of subgroup IIIa (single PRP-treated) revealed a partial improvement in the ultrastructure of their cells. Sertoli cells showed a few cytoplasmic vacuoles (Figure 14a). Type A spermatogonium showed a normal oval nucleus with mild dilated perinuclear space in some areas, and a few vacuoles in between the cells (Figure 14b). Concerning the primary spermatocytes, they had more or less normal mitochondria and a few cytoplasmic vacuoles (Figure 14c). A few rounded spermatids showed normal nuclei with acrosomal caps, but there was a mild discontinuity in their nuclear membrane. The cytoplasm showed the Golgi apparatus, dilated RER, and some swollen mitochondria (Figure 14d). A few middle and principal pieces with excess residual cytoplasm were found (Figure 14e).

**Figure 15:**
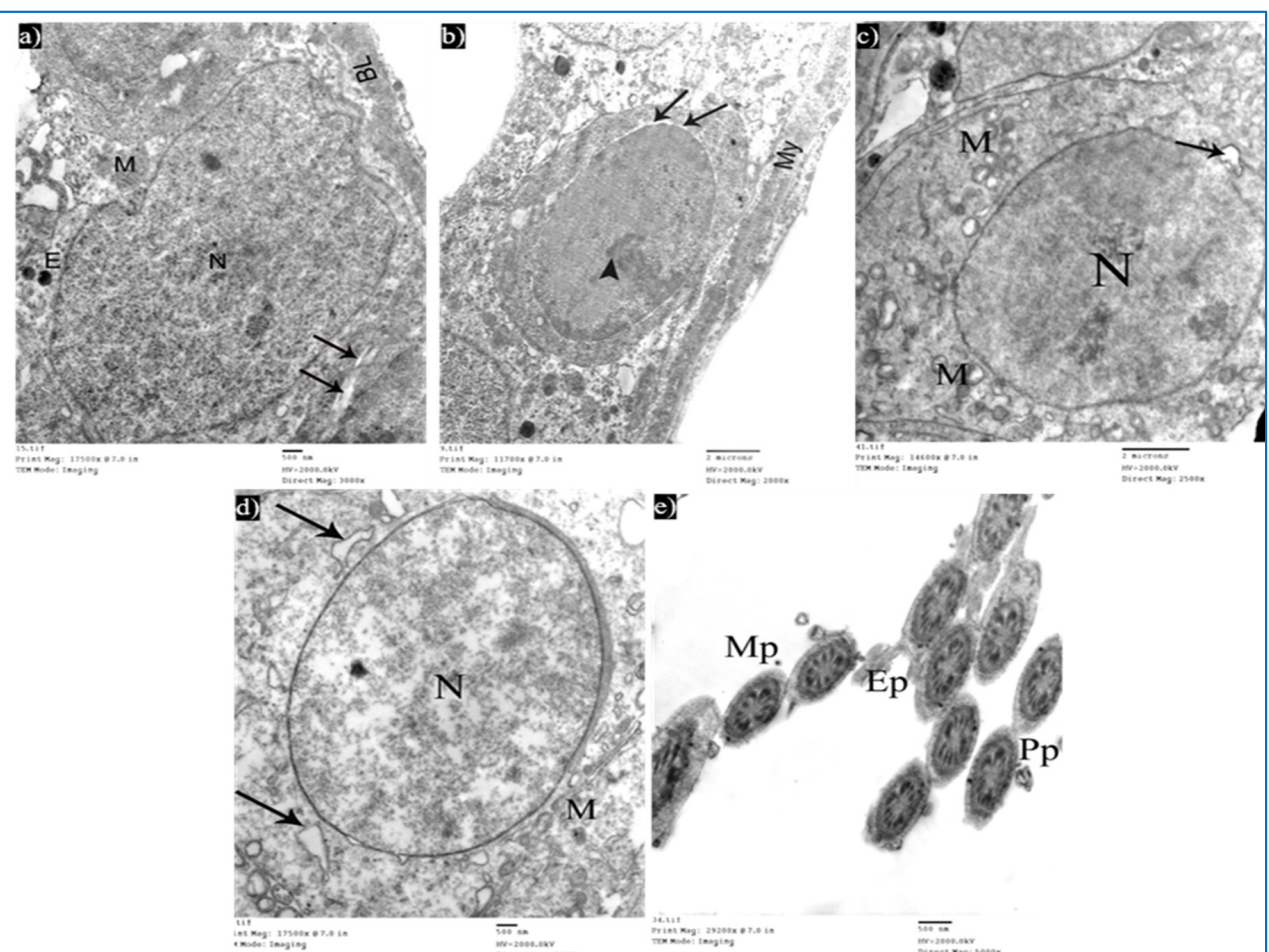
Electron micrographs of a rat of subgroup IIIb, showing: (**a**) A Sertoli cell with nucleus (N), mitochondria (M), and basal lamina (BL). Mic. Mag. 3000×. (**b**) Type A spermatogonium, nucleus with areas of heterochromatin (arrowhead) and focal perinuclear dilatation (arrows), and myoid cell (My). Mic. Mag. 2000×. (**c**) Primary spermatocyte, nucleus (N), mitochondria (M), and nuclear membrane shows localized dilatation (arrow). Mic. Mag. 2500×. (**d**) Round spermatid, normal nucleus (N), intact mitochondria (M), and dilated RER (arrows). Mic. Mag. 3000×. (**e**) Intact middle pieces (MP), principal pieces (PP), and end pieces (EP). Mic. Mag. 5000×.

Examination of the ultrathin sections of subgroup IIIb showed the normal histological structure of Sertoli and spermatogenic cells. Sertoli cells appeared with normal mitochondria (Figure 15a). A few spermatogonia revealed mild perinuclear dilatation and normal cytoplasm (Figure 15b). Primary spermatocytes showed normal cytoplasm and characteristic mitochondria, while in other cells, a focal dilatation in their nuclear membrane was noticed (Figure 15c). Early rounded spermatids appeared with normal-shaped nuclei, having fine chromatin and acrosomal caps. Normal mitochondria and dilated RER were also observed (Figure 15d). Intact transverse sections of spermatozoa tails were seen (Figure 15e).

#### 3.1.5. Morphometric Study and Statistical Results

The body weight after the administration of oxymetholone showed a noteworthy increase (at a *p*-value significance threshold of 0.001), as compared with the reference control group. Additionally, all experimental data showed reasonable normality as compared with the reference control group based on homogeneity of variance (all F values were lower than F critical). Furthermore, a nonsignificant difference was recorded between oxymetholone with or without PRP groups. All these data are illustrated in Table 2, and Appendix A and Appendix B.

The administration of oxymetholone caused a significant decrease in testis weight as compared with other groups. There was no significant difference regarding testis weight among PRP-treated groups (III) and between them and the control group. All data are analyzed in Table 2.

Statistical analysis of the data collected by the image analysis software program (Image J, version 1.48) revealed a nonsignificant difference between the control subgroups regarding epithelial height. The oxymetholone group revealed a highly significant decrease in the epithelial height when compared with the control group and PRP-treated groups, while the PRP groups revealed a nonsignificant difference in the epithelial height between each other and when compared with the control group. All these data are illustrated in Table 2. 

## 4. Discussion

Oxymetholone is one of the anabolic steroids prescribed by healthcare providers to treat hormonal-related problems, such as delayed puberty. Oxymetholone can also treat diseases that cause muscle loss, such as AIDS and cancer. However, some athletes and bodybuilders misuse this drug to improve their physical appearance. It has hazardous effects on health and fertility [30].

The high mitotic activity rate and the highly sensitive cellular composition of the spermatogenic epithelium make the testes more affected by occupational and environmental hazards than other tissues [31].

Platelet-rich plasma has attracted the attention of researchers due to its effect on accelerating and stimulating the healing process, as it contains a unique composition of growth factors and cytokines [21]. Its beneficial effect in the ischemic-reperfusion testicular model [18] and the testicular torsion model [19] has been confirmed. Therefore, this work aimed to investigate its therapeutic potential in oxymetholone-induced toxicity.

In the present study, there was a significant increase in body weight in the oxymetholone group (group II) as compared to the other groups. This was in accordance with Wang et al. [2], who detected an increase in body weight with oxymetholone treatment and explained this increase as oxymetholone increases muscle bulk, protein anabolism, and food intake. Regarding testicular weight, there was a decrease in the weight of the testes in group II. Akbari et al. [5] explained a similar finding by the occurrence of atrophy of the seminiferous tubules. 

In the current study, the oxymetholone-treated group revealed irregularities in the outline of the seminiferous tubules, discontinuity, and detachment from the overlying germinal epithelium in H&E- and PAS-stained sections. These irregularities may be due to tubular shrinkage of the degenerated seminiferous tubules [32]. Moreover, vacuolated cytoplasm of Sertoli cells, spermatogonia, and primary spermatocytes was detected. These findings were in accordance with previous studies [5,33]. These changes may be due to oxymetholone-induced oxidative stress, which increases reactive oxygen species (ROS) production, with consequent damage to their cell membranes as well as the membranes of cell organelles, thus increasing their permeability [34]. Kumar et al. [35] suggested that vacuoles within their cytoplasm represent pinched-off and distended segments of the endoplasmic reticulum. Additionally, they added that these vacuoles may be due to vacuolar degeneration or hydropic changes.

In this study, dilated congested blood vessels appeared between the tubules in the oxymetholone-treated group. This finding was attributed to oxymetholone toxicity [4,36]. Additionally, basal insertion of sperm in between spermatogenic cells may be due to rapid disruption of the junctions between Sertoli and germ cells [37]. Some authors attributed this to the change in the proportion of myoid cells and collagen fibers, which may hinder the proper spermatozoa release into the lumen [38]. 

Moreover, the height of the germinal epithelium significantly decreased as compared to the control group. Some seminiferous tubules contained only a few spermatozoa in their lumen. This was in accordance with Wang et al. [2], who suggested that the negative feedback mechanism of oxymetholone on the hypothalamic gonadal axis leads to a decrease in gonadotropin-releasing hormones and defects in the process of spermatogenesis, with a decreased number of mature sperms. Moreover, desquamated spermatogenic cells were present in the lumen of tubules, which may be due to the destruction of the cellular processes of Sertoli cells that are present between the germ cells, leading to exfoliation of the spermatogenic cells into the lumen of the seminiferous tubules [5].

In this work, there were multiple abnormalities in sperm morphology, either head or tail abnormalities, in the oxymetholone-treated group. Wang et al. [2] also detected abnormal shapes of sperm after using oxymetholone. They explained that these changes may be due to the defect in the process of spermatogenesis caused by ROS formation. 

The electron microscopic examination of the oxymetholone-treated group showed dilated SER and cytoplasmic vacuolations affecting Sertoli cells. These results denoted degeneration of Sertoli cells, or they may be due to the autophagosome formation of the necrotic germ cells [39]. Furthermore, some swollen mitochondria were present in the cytoplasm of Sertoli cells, primary spermatocytes, and early spermatids. These results can be explained by the oxidative stress that led to the opening of the mitochondrial permeability transition pore (PTP), which causes the formation of a high-conductance channel in the inner mitochondrial membrane that causes mitochondrial swelling [40].

Additionally, many heterogeneous electron-dense bodies were noticed in the cytoplasm of Sertoli cells, primary spermatocytes, and early rounded spermatids. These findings can be caused by the autophagy of damaged cytoplasmic debris [41]. Kumar et al. [35] explained that a moderate increase in the number of autophagosomes might be explained as a defense mechanism that allows the elimination of different parts of the cytoplasm. Moreover, the middle pieces of spermatozoa tails showed an excess retained cytoplasm, which may be due to spermiogenesis arrest and cytoplasmic extrusion interruption [34].

In this work, the PRP injection to oxymetholone-induced testicular injury in adult albino rats resulted in a significant increase in the testes weight. Additionally, the PRP injection improved the histological architecture of the testes and helped in the normal process of spermatogenesis. This was proven by the significant increase in the height of the germinal epithelium, as compared with the oxymetholone group. The improvement of testicular damage by PRP was due to the presence of many growth factors and cytokines, such as insulin growth factor-1 (IGF-1), which has a vital role in tissue repair. It can overcome testicular damage by increasing the synthesis of antioxidant enzymes [18].

Thus, PRP reduces oxidative stress and reactive oxygen species that help decrease vacuolations and degenerations. Platelet-derived growth factor (PDGF) has an important role in the regulation of autocrine/paracrine and germinal cell function. Additionally, VEGF regulates microenvironmental changes in testicular tissue through a paracrine mechanism. VEGF helps to stimulate the testicular epithelial cells’ proliferation and maintain testicular microcirculation permeability [42].

These growth factors lessen tissue ischemia of the testes and help in germinal epithelium maintenance and Sertoli and Leydig cells’ function regulation [21].

Injection of PRP can reduce oxidative stress, MDA (malondialdehyde, an oxidative marker), and IL-6 (a pro-inflammatory factor) levels in the testes of rats. Additionally, it was suggested that PRP repaired testicular damage caused by lipid peroxidation processes by increasing the biosynthesis of antioxidant enzymes such as catalase and superoxide dismutase [43].

Concerning mammalian testes, they are highly sensitive to oxidative stress due to the abundant unsaturated fatty acids. Oxidative stress is defined as an imbalance between the systemic manifestation of reactive oxygen species (ROS) and the antioxidant capacity of the body. Normally, ROS levels are low and controlled by antioxidant systems. Their presence is important for many processes, such as capacitation, acrosome reaction, hyperactivation, spermatozoa–oocyte fusion, DNA damage, protein denaturation, and apoptosis [44]. Cellular proliferation and apoptosis are basic life phenomena that are necessary measures to maintain the dynamic balance of cell numbers in the body. Apoptosis is an orderly death process regulated by genes that play a vital role in maintaining the internal environment of the body. Oxidative stress also changes the intracellular concentration of Ca^2+^ in spermatozoa and causes sperm apoptosis, as marked elevation of Ca^2+^ stimulates hydrolytic enzymes, causing an exaggeration in energy expenditure, an impairment in energy production, and resulting in cell death [45].

Additionally, ROS regulates the expression of apoptosis-related genes (Bax and Bcl-2), which ultimately leads to cytochrome-c leakage from mitochondria. Cytochrome-c binds to the apoptotic protease-activating factor 1, forming apoptosome complexes that activate caspases and induce apoptosis. The activation of necrosis factor kappa beta (NFkβ) due to oxidative stress causes an increase in the production of pro-inflammatory cytokines, such as interleukin-6 (IL-6). Moreover, an increase in the levels of MDA and IL-6 in the testes causes testicular damage [46].

Growth factors in PRP play a major role in tissue regeneration, having biological effects as anti-inflammatory agents and antioxidants. Previous research has concluded that vascular endothelial growth factor (VEGF) is important for the survival of spermatogonia stem cells, induces germ cell proliferation, maintains their lifecycle, and inhibits germ cell apoptosis [47]. Studies have shown that VEGF regulates microenvironmental changes in testicular tissue through a paracrine mechanism. VEGF helps to maintain testicular microcirculation permeability and stimulates the proliferation of testicular epithelial cells [48]. Insulin growth factor-1 (IGF-1) also has an improving effect on spermatogenesis and steroidogenesis. It also plays a role in the biosynthesis of antioxidant enzymes [49]. Epidermal growth factor (EGF) has a beneficial effect on spermatogenesis. Platelet-derived growth factor (PDGF) has a positive role in the germinal cells and autocrine/paracrine function regulation. These growth factors reduce the tissue ischemia of the testes, help in the maintenance of germinal epithelium, as well as in regulating Sertoli and Leydig cells’ function. It seems that the combination of these growth factors might have a positive effect on spermatogenesis and fertility [50]. Additionally, Ferreira-Dos-Santos et al. [51] attributed these bio-regenerative actions to enhancing the secretion of anti-inflammatory factors, angiogenic factors, and proteins concerned with extracellular matrix remodeling. Previous studies have shown the effect of PRP on oxidative stress in many organs. Rizal et al. [52] studied the effect of PRP on oxidative stress on the testis, Martins et al. [53] on skeletal muscle, Hudgen et al. [54] on the tendon, Soliman et al. [55] on the kidney, and Zarin et al. [56] on the pancreas. Based on these studies, PRP has promising effects in decreasing inflammatory effects and oxidative stress induced by various toxic agents.

During this study, treatment of the oxymetholone group with PRP once (subgroup IIIa) led to a mild improvement in the histological structure of the testis in the form of an apparent reduction in the interstitial tissue spaces, a decrease in the vacuolations and intercellular spaces, as well as an increase in sperm density in the lumen of the tubules. Additionally, there were dilated congested blood vessels in the interstitial tissue, with mild separation of the basal lamina of a few tubules. A few Leydig cells in the interstitial spaces with little vacuolated acidophilic exudate in between were still observed. 

In this research, rats that were treated with PRP twice in subgroup IIIb showed preservation of the histological structure of the testis to a great extent. The improvement was better than that of subgroup IIIa, which was in the form of regeneration and proliferation of the spermatogenic epithelium, nearly absent vacuolations, no separation of basal lamina, in addition to the presence of many mature sperms in the lumen. On the ultrastructural level, there was an improvement in the form of decreased perinuclear spaces, normal spermatogenic and Sertoli cells, regular acrosomal caps and nuclear membrane of early spermatid, and normal sperm heads, tails, as well as Leydig cells. Similarly, the authors of [43,57] established that repeated doses of PRP had an ameliorating effect on the induced testicular damage.

This research showed that multiple intratesticular PRP injections after oxymetholone injury yielded better results than single injections with PRP. Some evidence suggested different results between single and multiple injections of PRP because most growth factors contained in platelets are short-lived [58,59]. 

## 5. Conclusions

From this study, it can be concluded that oxymetholone caused structural changes in the testes of adult albino rats and induced multiple sperm morphology abnormalities, suggesting male infertility. PRP renews the testis microstructure and causes regeneration of the tubules as it contains multiple growth factors and cytokines. Injecting PRP twice yielded better results in the restoration of the testicular architecture than the single injection because most growth factors contained in platelets are short-lived. It is recommended that local injection of PRP is a promising therapeutic regimen aiming to restore spermatogenesis in oxymetholone toxicity.

## Figures and Tables

**Table 1:** Animal groups.

Subgroup	Number of Rats	Maneuvers	Time of Sacrifice
Group 0			
	10 rats	Donation of blood	At the time of donation
Group I			
Subgroup Ia	5 rats	Left untreated.	At the end of the experiment
Subgroup Ib	5 rats	PRP injection (0.1 mL, intratesticular, once)	At the time of the sacrifice of the related subgroup IIIa
Subgroup Ic	5 rats	PRP injection (twice, with two-week intervals between each injection).	At the time of the sacrifice of the related subgroup IIIb
Group II			
	8 rats	Oxymetholone (10 mg/kg) for 30 days.	After 30 days
Group III			
Subgroup IIIa	8 rats	Oxymetholone + PRP injection, once, after oxymetholone.	After 6 weeks from the start of the experiment
Subgroup IIIb	8 rats	Oxymetholone + PRP injection, twice, two-week intervals, after oxymetholone.	After 8 weeks from the start of the experiment

**Table 2:** The changes in the body weight, testis weight, and height of the germinal epithelium in different groups.

		Unit	Ia (a)	II (b)	IIIa (c)	IIIb (d)	*p*-Value Significance Threshold
Body weight	Range	g	200–260	250–295	265–300	270–310	0.001
	Mean ± SD		219.00 ± 24.08 ^b,c,d^	267.90 ± 18.01 ^a^	281.50 ± 12.97 ^a^	285.32 ± 16.37 ^a^	
Body weight change	Range	g	40.07–98.06	90.52–133.72	103.45–138.14	111.46–151.02	
	Mean ± SD		59.34 ± 22.98 ^b,c,d^	107.49 ± 17.51 ^a^	120.92 ± 12.78 ^a^	126.48 ± 16.28 ^a^	
Testis weight	Range	g	1.37–1.63	1–1.25	1.2–1.4	1.25–1.4	
	Mean ± SD		1.51 ± 0.10 ^b,c,d^	1.15 ± 0.10 ^a^	1.28 ± 0.09 ^a^	1.32 ± 0.06 ^a^	
Height of germinal epithelium	Range	μm	92.55–105.54	49.55–58.25	71.57–76.93	84.56–90.55	
	Mean ± SD		98.70 ± 4.98 ^b,c,d^	53.68 ± 3.95 ^a^	74.97 ± 2.27 ^a^	86.56 ± 2.37 ^a^	

Significance is indicated by superscript letters ^a^, ^b^, ^c^, and ^d^, against groups Ia, II, IIIa, and IIIb, respectively.

## Data Availability

Not applicable.

## Appendix A

**Table A1:** Measured data and their statistical analysis.

	Body Weight				Body Weight Change				Testis Weight				Height of Germinal Epithelium			
	Ia	II	IIIa	IIIb	Ia	II	IIIa	IIIb	Ia	II	IIIa	IIIb	Ia	II	IIIa	IIIb
	200	250.03	265.02	292.23	40.07	90.52	103.45	133.9	1.37	1.01	1.2	1.25	92.554	49.55	71.57	85.16
	260	295.01	300.03	310.01	98.06	133.72	138.14	151.02	1.5	1.08	1.22	1.32	95.385	50.48	73.77	84.56
	210	276.21	286.7	282.12	50.96	115.77	126.66	122.14	1.6	1.25	1.37	1.38	99.75	52.75	75.95	85.76
	220	262.42	279.4	272.25	61.27	102.37	119.71	113.87	1.63	1.23	1.4	1.4	100.27	58.25	76.65	86.77
	205	255.85	276.35	270.02	46.34	95.08	116.67	111.4	1.47	1.21	1.21	1.29	105.54	57.35	76.93	90.55
Mean	219	267.90	281.5	285.32	59.34	107.49	120.92	126.48	1.51	1.15	1.28	1.32	98.70	53.67	74.97	86.56
Standard deviation	24.08319	18.01	12.97	16.37	22.98	17.51	12.78	16.28	0.10	0.10	0.09	0.06	4.97	3.95	2.27	2.37
Minimum	200	250.03	265.02	270.02	40.07	90.52	103.45	111.46	1.37	1.01	1.2	1.25	92.55	49.55	71.57	84.56
Maximum	260	295.01	300.03	310.01	98.06	133.72	138.14	151.02	1.63	1.25	1.4	1.4	105.54	58.25	76.93	90.55

## Appendix B

**Table A2:** *t*-Test and F-test for comparisons of two groups.

	Body Weight					
	Ia and II		Ia and IIIa		Ia and IIIb	
Mean	219	267.904	219	281.5	219	285.326
Variance	580	324.644	580	168.231	580	268.12473
Observations	5	5	5	5	5	5
Pearson Correlation	0.886938		0.87721		0.70427824	
t Stat	−9.41474		−9.87703		−8.6693657	
P (T ≤ t) one-tail	0.000355		0.000295		0.00048708	
t Critical one-tail	2.131847		2.131847		2.13184679	
P (T ≤ t) two-tail	0.000709		0.00059		0.00097416	
t Critical two-tail	2.776445		2.776445		2.77644511	
F	1.78		3.44		2.16	
F Critical one-tail	6.38		6.38		6.38	
	**Testis Weight**					
	**Ia and II**		**Ia and IIIa**		**Ia and IIIb**	
Mean	1.514	1.156	1.514	1.28	1.514	1.328
Variance	0.01093	0.01108	0.01093	0.00935	0.01093	0.00387
Observations	5	5	5	5	5	5
Pearson Correlation	0.826462		0.917483		0.99326917	
t Stat	12.95199		12.57992		9.59221759	
P (T ≤ t) one-tail	0.000102		0.000115		0.00033008	
t Critical one-tail	2.131847		2.131847		2.13184679	
P (T ≤ t) two-tail	0.000205		0.00023		0.00066015	
t Critical two-tail	2.776445		2.776445		2.77644511	
F	1.01		1.16		2.82	
F Critical one-tail	6.38		6.38		6.38	
	**Height of Germinal Epithelium**					
	**Ia and II**		**Ia and IIIa**		**Ia and IIIb**	
Mean	98.7008	53.676	98.7008	74.974	98.7008	86.56
Variance	24.78282	15.62888	24.78282	5.15608	24.7828157	5.64105
Observations	5	5	5	5	5	5
Pearson Correlation	0.855092		0.92789		0.88839717	
t Stat	38.73921		17.72335		8.8473015	
P (T ≤ t) one-tail	1.33 × 10^−6^		2.98 × 10^−5^		0.00045057	
t Critical one-tail	2.131847		2.131847		2.13184679	
P (T ≤ t) two-tail	2.65 × 10^−6^		5.95 × 10^−5^		0.00090115	
t Critical two-tail	2.776445		2.776445		2.77644511	
F	1.58		4.80		4.39	
F Critical one-tail	6.38		6.38		6.38	

## References

[1] Rim K.-T. (2017). Reproductive Toxic Chemicals at Work and Efforts to Protect Workers’ Health: A Literature Review. Saf. Health Work.

[2] Wang Y., Bai L., Li H., Yang W., Li M. (2021). Protective Effects of *Lepidium draba* L. Leaves Extract on Testis Histopathology, Oxidative Stress Indicators, Serum Reproductive Hormones and Inflammatory Signalling in Oxymetholone-treated Rat. Andrologia.

[3] Mehrpour O., Nakhaee S., Barangi S., Karimi G.B.T.-R.M. (2022). Reference Module in Biomedical Sciences.

[4] Feng J., Gao H., Yang L., Xie Y., El-Kenawy A.E., El-kott A.F. (2022). Renoprotective and Hepatoprotective Activity of *Lepidium draba* L. Extracts on Oxymetholone-induced Oxidative Stress in Rat. J. Food Biochem..

[5] Akbari Bazm M., Goodarzi N., Shahrokhi S.R., Khazaei M. (2020). The Effects of Hydroalcoholic Extract of *Vaccinium arctostaphylos* L. on Sperm Parameters, Oxidative Injury and Apoptotic Changes in Oxymetholone-induced Testicular Toxicity in Mouse. Andrologia.

[6] Attia S., Narberhaus C., Schaaf H., Streckbein P., Pons-Kühnemann J., Schmitt C., Neukam F.W., Howaldt H.-P., Böttger S. (2020). Long-Term Influence of Platelet-Rich Plasma (PRP) on Dental Implants after Maxillary Augmentation: Retrospective Clinical and Radiological Outcomes of a Randomized Controlled Clinical Trial. J. Clin. Med..

[7] Pochini A.d.C., Antonioli E., Bucci D.Z., Sardinha L.R., Andreoli C.V., Ferretti M., Ejnisman B., Goldberg A.C., Cohen M. (2016). Analysis of Cytokine Profile and Growth Factors in Platelet-Rich Plasma Obtained by Open Systems and Commercial Columns. Einstein.

[8] Bos-Mikich A., de Oliveira R., Frantz N. (2018). Platelet-Rich Plasma Therapy and Reproductive Medicine. J. Assist. Reprod. Genet..

[9] Ozcan P., Takmaz T., Tok O.E., Islek S., Yigit E.N., Ficicioglu C. (2020). The Protective Effect of Platelet-Rich Plasma Administrated on Ovarian Function in Female Rats with Cy-Induced Ovarian Damage. J. Assist. Reprod. Genet..

[10] Hosseinisadat R., Farsi Nejad A., Mohammadi F. (2023). Intra-Ovarian Infusion of Autologous Platelet-Rich Plasma in Women with Poor Ovarian Reserve: A before and after Study. Eur. J. Obstet. Gynecol. Reprod. Biol..

[11] Sabouni R., Tarrab R., Kalaji D., Abbassi H. (2022). A New Approach of Using Platelet-Rich Autologous Plasma to Increase the Ovarian Reservoir in a Syrian Patient with Ovarian Insufficiency: A Case Report. Ann. Med. Surg..

[12] Bigliardi E., Cantoni A.M., De Cesaris V., Denti L., Conti V., Bertocchi M., Di Ianni F., Parmigiani E., Grolli S. (2018). Use of Platelet-Rich Plasma for the Treatment of Prostatic Cysts in Dogs. Can. J. Vet. Res..

[13] Wu Y.-N., Liao C.-H., Chen K.-C., Chiang H.-S. (2021). CXCL5 Cytokine Is a Major Factor in Platelet-Rich Plasma’s Preservation of Erectile Function in Rats After Bilateral Cavernous Nerve Injury. J. Sex. Med..

[14] Rezaei M., Badiei R., Badiei R. (2019). The Effect of Platelet-Rich Plasma Injection on Post-Internal Urethrotomy Stricture Recurrence. World J. Urol..

[15] Akhoundova F., Schumacher F., Léger M., Berndt S., Martinez de Tejada B., Abdulcadir J. (2022). Use of Autologous Platelet Rich Plasma (A-PRP) for Postpartum Perineal Repair Failure: A Case Report. J. Pers. Med..

[16] Everts P.A., Mazzola T., Mautner K., Randelli P.S., Podesta L. (2022). Modifying Orthobiological PRP Therapies Are Imperative for the Advancement of Treatment Outcomes in Musculoskeletal Pathologies. Biomedicines.

[17] Le V.-T., Nguyen Dao L.T., Nguyen A.M. (2022). Transforaminal Injection with Autologous Platelet-Rich Plasma in Lumbar Disc Herniation: A Single-Center Prospective Study in Vietnam. Asian J. Surg..

[18] Sekerci C.A., Tanidir Y., Sener T.E., Sener G., Cevik O., Yarat A., Alev-Tuzuner B., Cetinel S., Kervancioglu E., Sahan A. (2017). Effects of Platelet-Rich Plasma against Experimental Ischemia/Reperfusion Injury in Rat Testis. J. Pediatr. Urol..

[19] Sekerci C.A., Tanidir Y., Sener T.E., Sahan A., Cevik O., Yarat A., Tuzuner B.A., Cetinel S., Demir E.K., Sener G. (2016). MP43-15 Protective Effect of Platelet Rich Plasma on Experimental Ischemia/Reperfusion Injury in Torsion of Rat Testis. J. Urol..

[20] Council N.R. (2010). Guide for the Care and Use of Laboratory Animals.

[21] Dehghani F., Sotoude N., Bordbar H., Panjeshahin M.R., Karbalay-Doust S. (2019). The Use of Platelet-Rich Plasma (PRP) to Improve Structural Impairment of Rat Testis Induced by Busulfan. Platelets.

[22] Abd Z.L. (2021). Effect of Oxymetholone on the Sperm Quality and Sex Hormone Profile in Male Rats. Indian J. Forensic Med. Toxicol..

[23] Samy A., El-Adl M., Rezk S., Marghani B., Eldomany W., Eldesoky A., Elmetwally M.A. (2020). The Potential Protective and Therapeutic Effects of Platelet-Rich Plasma on Ischemia/Reperfusion Injury Following Experimental Torsion/Detorsion of Testis in the Albino Rat Model. Life Sci..

[24] Kondracki S., Wysokińska A., Kania M., Górski K. (2017). Application of Two Staining Methods for Sperm Morphometric Evaluation in Domestic Pigs. J. Vet. Res..

[25] Suvarna K.S., Layton C., Bancroft J.D. (2018). Bancroft’s Theory and Practice of Histological Techniques E-Book.

[26] Wulff S., Hafer L., Cheles M., Couture R., Holliday J.M., Smith S., Stanforth D.A. (2004). Guide to Special Stains.

[27] Goodhew P.J. (2020). Specimen Preparation for Transmission Electron Microscopy of Materials.

[28] Davies H.T.O., Crombie I.K. (2009). What Are Confidence Intervals and P-Values.

[29] Oberlender G., Murgas L.D.S., Zangerônimo M.G., Silva A.C., Pereira L.J., Muzzi R.A.L. (2012). Comparison of Two Different Methods for Evaluating Boar Semen Morphology. Arch. Med. Vet..

[30] Tauchen J., Jurášek M., Huml L., Rimpelová S. (2021). Medicinal Use of Testosterone and Related Steroids Revisited. Molecules.

[31] Soliman A.H.M., Ibrahim I.A., Shehata M.A., Mohammed H.O. (2020). Histopathological and Genetic Study on the Protective Role Of-Carotene on Testicular Tissue of Adult Male Albino Rats Treated with Titanium Dioxide Nanoparticles. Afr. J. Pharm. Pharmacol..

[32] Mohamed D.A., Abdelrahman S.A. (2019). The Possible Protective Role of Zinc Oxide Nanoparticles (ZnONPs) on Testicular and Epididymal Structure and Sperm Parameters in Nicotine-Treated Adult Rats (a Histological and Biochemical Study). Cell Tissue Res..

[33] Najafi G., Nejati V., Shalizar Jalali A., Zahmatkesh E. (2014). Protective Role of Royal Jelly in Oxymetholone-Induced Oxidative Injury in Mouse Testis. Iran. J. Toxicol..

[34] Ahmed M.A.E. (2015). Amelioration of Nandrolone Decanoate-Induced Testicular and Sperm Toxicity in Rats by Taurine: Effects on Steroidogenesis, Redox and Inflammatory Cascades, and Intrinsic Apoptotic Pathway. Toxicol. Appl. Pharmacol..

[35] Kumar V., Abbas A.K., Aster J.C. (2017). Robbins Basic Pathology E-Book.

[36] Amirkhani R., Farzaei M.H., Ghanbari E., Khazaei M., Aneva I. (2022). Cichorium Intybus Improves Hepatic Complications Induced by Oxymetholone: An Animal Study. J. Med. Plants By-Prod..

[37] Richburg J.H. (2000). The Relevance of Spontaneous-and Chemically-Induced Alterations in Testicular Germ Cell Apoptosis to Toxicology. Toxicol. Lett..

[38] El Shafai A., Zohdy N., El Mulla K., Hassan M., Morad N. (2011). Light and Electron Microscopic Study of the Toxic Effect of Prolonged Lead Exposure on the Seminiferous Tubules of Albino Rats and the Possible Protective Effect of Ascorbic Acid. Food Chem. Toxicol..

[39] Ahmed R.R., Abdul-Hamid M., Galaly S.R., Hamdalla H.M. (2019). Monosodium Glutamate-Induced Liver Microscopic and Biochemical Changes in Male Rats, and the Possible Amendment of Quercetin. Egypt. J. Zool..

[40] Mansour A.M., Ibrahim M.A., Laag E.M., Zamzam A.F. (2018). Histological Study of the Effect of Semicarbazide on Testicular Seminiferous Tubules of Juvenile Albino Rat. Ain Shams J. Forensic Med. Clin. Toxicol..

[41] El-Beltagi E.M., Elwan W.M., El-Bakry N.A.M., Salah E.F. (2017). Histological and Immunohistochemical Study on the Effect of Gibberellic Acid on the Seminiferous Tubules of Testis of Adult Albino Rat and the Possible Protective Role of Grape Seeds Proanthocyanidin Extract. Tanta Med. J..

[42] Bakacak M., Bostanci M.S., İnanc F., Yaylali A., Serin S., Attar R., Yildirim G., Yildirim O.K. (2016). Protective Effect of Platelet Rich Plasma on Experimental Ischemia/Reperfusion Injury in Rat Ovary. Gynecol. Obstet. Investig..

[43] Hermilasari R.D., Rizal D.M., Wirohadidjojo Y.W. (2020). Effect of Platelet-Rich Plasma (PRP) on Testicular Damage in Streptozotocin-Induced Diabetic Rats. Bali Med. J..

[44] Bader R., Ibrahim J.N., Moussa M., Mourad A., Azoury J., Azoury J., Alaaeddine N. (2020). In Vitro Effect of Autologous Platelet-rich Plasma on H2O2-induced Oxidative Stress in Human Spermatozoa. Andrology.

[45] Cong M., Xu H., Li Y., Tian W., Lv J. (2022). Modifications of Calcium Metabolism and Apoptosis after Ammonia Nitrogen Exposure Imply a Tumorous Fate in Clam Ruditapes Philippinarum?. Aquat. Toxicol..

[46] Zhang J., Liu J., Ren L., Wei J., Duan J., Zhang L., Zhou X., Sun Z. (2018). PM2. 5 Induces Male Reproductive Toxicity via Mitochondrial Dysfunction, DNA Damage and RIPK1 Mediated Apoptotic Signaling Pathway. Sci. Total Environ..

[47] Caires K.C., De Avila J.M., Cupp A.S., McLean D.J. (2012). VEGFA Family Isoforms Regulate Spermatogonial Stem Cell Homeostasis in Vivo. Endocrinology.

[48] Xing J., Yu G., Xiang Y., Xu H., Liu Z., Bai Z. (2022). Effect of Low Energy Shock Wave on Testicular Microenvironment Homeostasis in Rats. Ecotoxicol. Environ. Saf..

[49] Griffeth R.J., Bianda V., Nef S. (2014). The Emerging Role of Insulin-like Growth Factors in Testis Development and Function. Basic Clin. Androl..

[50] Kutluhan M.A., Özsoy E., Şahin A., Ürkmez A., Topaktaş R., Toprak T., Gümrükçü G., Verit A. (2021). Effects of Platelet-rich Plasma on Spermatogenesis and Hormone Production in an Experimental Testicular Torsion Model. Andrology.

[51] Ferreira-Dos-Santos G., Hurdle M.F.B., Clendenen S.R., Eldrige J.S., Qu W. (2022). Autologous Platelet-Rich Plasma Applications in Chronic Pain Medicine: Establishing a Framework for Future Research-A Narrative Review. Pain Physician.

[52] Rizal D.M., Puspitasari I., Yuliandari A. Protective Effect of PRP against Testicular Oxidative Stress on D-Galactose Induced Male Rats. Proceedings of the 6th International Conference on Biological Science ICBS 2019: “Biodiversity as a Cornerstone for Embracing Future Humanity”.

[53] Martins R.P., Hartmann D.D., de Moraes J.P., Soares F.A.A., Puntel G.O. (2016). Platelet-Rich Plasma Reduces the Oxidative Damage Determined by a Skeletal Muscle Contusion in Rats. Platelets.

[54] Hudgens J.L., Sugg K.B., Grekin J.A., Gumucio J.P., Bedi A., Mendias C.L. (2016). Platelet-Rich Plasma Activates Proinflammatory Signaling Pathways and Induces Oxidative Stress in Tendon Fibroblasts. Am. J. Sport. Med..

[55] Soliman A.F., Saif-Elnasr M., Fattah S.M.A. (2019). Platelet-Rich Plasma Ameliorates Gamma Radiation-Induced Nephrotoxicity via Modulating Oxidative Stress and Apoptosis. Life Sci..

[56] Zarin M., Karbalaei N., Keshtgar S., Nemati M. (2019). Platelet-Rich Plasma Improves Impaired Glucose Hemostasis, Disrupted Insulin Secretion, and Pancreatic Oxidative Stress in Streptozotocin-Induced Diabetic Rat. Growth Factors.

[57] Sayed W.M., Elzainy A. (2021). Impact of Platelet-Rich Plasma versus Selenium in Ameliorating Induced Toxicity in Rat Testis: Histological, Immunohistochemical, and Molecular Study. Cell Tissue Res..

[58] Zayni R., Thaunat M., Fayard J.-M., Hager J.-P., Carrillon Y., Clechet J., Gadea F., Archbold P., Cottet B.S. (2015). Platelet-Rich Plasma as a Treatment for Chronic Patellar Tendinopathy: Comparison of a Single versus Two Consecutive Injections. Muscles. Ligaments Tendons J..

[59] Chouhan D.K., Dhillon M.S., Patel S., Bansal T., Bhatia A., Kanwat H. (2019). Multiple Platelet-Rich Plasma Injections versus Single Platelet-Rich Plasma Injection in Early Osteoarthritis of the Knee: An Experimental Study in a Guinea Pig Model of Early Knee Osteoarthritis. Am. J. Sport. Med..

